# Capturing systematically users' experience of evaluation tools for integrated AMU and AMR surveillance

**DOI:** 10.3389/fvets.2023.1107122

**Published:** 2023-03-24

**Authors:** Lis Alban, Marion Bordier, Barbara Häsler, Lucie Collineau, Laura Tomassone, Houda Bennani, Cécile Aenishaenslin, Madelaine Norström, Maurizio Aragrande, Maria Eleni Filippitzi, Pedro Moura, Marianne Sandberg

**Affiliations:** ^1^Department for Food Safety, Veterinary Issues and Risk Analysis, Danish Agriculture and Food Council, Copenhagen, Denmark; ^2^Department of Veterinary and Animal Sciences, University of Copenhagen, Frederiksberg, Denmark; ^3^ASTRE, Université de Montpellier, CIRAD, INRAE, Montpellier, France; ^4^Laboratoire National de l'Elevage et de Recherches Vétérinaires, Institut Sénégalais de Recherches Agricoles, Dakar, Senegal; ^5^CIRAD, UMR ASTRE, Dakar, Senegal; ^6^Veterinary Epidemiology Economics and Public Health Group, Department of Pathobiology and Population Sciences, Royal Veterinary College, London, United Kingdom; ^7^University of Lyon, French Agency for Food, Environmental and Occupational Health & Safety (ANSES), Laboratory of Lyon, Epidemiology and Surveillance Support Unit, Lyon, France; ^8^Department of Veterinary Sciences, University of Turin, Grugliasco (Turin), Italy; ^9^Département de pathologie et microbiologie, Université de Montréal, Saint-Hyacinthe, QC, Canada; ^10^Department of Animal Health, Welfare and Food Safety, Norwegian Veterinary Institute, Ås, Norway; ^11^Department of Agricultural and Food Sciences, University of Bologna, Bologna, Italy; ^12^Laboratory of Animal Health Economics, Aristotle University of Thessaloniki, Thessaloniki, Greece; ^13^Veterinary Epidemiology Unit, Sciensano, Brussels, Belgium; ^14^National Food Institute, Technical University of Denmark, Lyngby, Denmark

**Keywords:** One Health assessment, integrated surveillance, evaluation, antimicrobial resistance, antimicrobial use

## Abstract

Tackling antimicrobial resistance (AMR) is a goal for many countries. Integrated surveillance of antimicrobial use (AMU) and resistance is a prerequisite for effective risk mitigation. Regular evaluation of any surveillance is needed to ensure its effectiveness and efficiency. The question is how to evaluate specifically integrated surveillance for AMU and AMR. In an international network called CoEvalAMR, we have developed guidelines for selection of the most appropriate tools for such an evaluation. Moreover, we have assessed different evaluation tools as examples using a country case format and a methodology with a focus on the user's experience. This paper describes the updated methodology, which consists of a brief introduction to the case and to the tool separately. Moreover, there are 12 functional aspects and nine content themes which should be scored using a 4-tiered scale. Additionally, four Strengths, Weaknesses, Opportunities, Threats (SWOT) questions should be addressed. Results are illustrated using radar diagrams. An example of application of the updated methodology is given using the ECoSur evaluation tool. No tool can cover all evaluation aspects comprehensively in a user-friendly manner, so the choice of tool must be based upon the specific evaluation purpose. Moreover, adequate resources, time and training are needed to obtain useful outputs from the evaluation. Our updated methodology can be used by tool users to share their experience with available tools, and hereby assist other users in identifying the most suited tool for their evaluation purpose. Additionally, tool developers can get valuable information for further improvements of their tool.

## Introduction

It is a common goal of society to keep antimicrobials effective for the coming generations. One way of supporting this goal is to have surveillance in place for antimicrobial use (AMU) and resistance in different domains and sectors. This should preferably be done in an integrated manner, because genes coding for antimicrobial resistance (AMR) are spread within and among different human, animal and environmental domains. To ensure surveillance effectiveness and efficiency, there is a need to evaluate existing surveillance systems or components at regular intervals (1). This will help to reach the objective of surveillance which, among others, is to determine why and where action is needed to modify AMU and hereby reduce AMR.

Several tools have been developed to assist in such evaluations. Evaluation may be done by different types of professionals, who may be acting internally or externally to the surveillance system under evaluation. The users will have varying levels of experience in surveillance evaluation, access to detailed data and time to dedicate to the evaluation. Moreover, evaluation may be pursued for different purposes. This makes it necessary to choose the right tool for a given evaluation context, team and question.

During 2019–2020, an international network of scientists called “Convergence in evaluation frameworks for integrated surveillance of AMU and AMR” (CoEvalAMR) developed guidance for the evaluation of integrated surveillance for AMU and AMR (2). In this network, we defined integrated surveillance of AMU and AMR in the context of One Health as surveillance that is based on a systemic, cross-sectoral, multi-stakeholder perspective to inform mitigation decisions with the aim to keep antimicrobials effective for future generations (https://guidance.fp7-risksur.eu/welcome/evaluation-of-surveillance/). In Phase 1 of the CoEvalAMR project, a methodology was developed to gather user feedback on evaluation tools for integrated surveillance for AMU and AMR in an easy and standardized way. The focus was on compiling user subjective experience on the application of the tools; the approach chosen was partly inspired by websites using user feedback and scoring to inform decision-making of other users. The methodology consisted of four different approaches that complemented each other. The first consisted of a brief description of the case study, whereas the second covered the assessment of 11 pre-defined functional aspects of the tool including workability regarding the need for data, time and people (Table 1). The third approach covered an assessment of seven predefined content themes related to the tools' scope (Table 2). The functional aspects and content themes were scored semi-quantitatively using a scale from 1 to 4, and a comment was requested explaining the score. The fourth approach consisted of the subjective perception of the tool assessors based on an assessment of the strengths, weaknesses, opportunities, and threats (SWOT) (Table 3).

**Table 1:** Description of the updated list of 12 functional aspects, sorted into five groups—text in bold reflects changes to the original methodology.

**Group of aspects**	**Functional aspect**	Scales and scores^*^
**1—Ease of use**	User-friendliness **related to wording, guidance and layout of the tool or framework**	**(1) Very difficult to use, (2) difficult to use, (3) manageable to use, (4) simple to use**
	Analysis and interpretation of evaluation data	**(1) Very difficult, (2) difficult, (3) manageable, (4) simple**
	Amount **and complexity** of data required, **where complexity is defined as different kinds of data from multiple sources in different formats or as primary data collection required**	**(1) High amount of complex data required, (2) moderate amount of complex data required, (3) low amount of complex data required, (4) simple kind of existing data required**
**2—Scope**	**Ability to address** the stated evaluation objectives	**(1) Not at all, (2) only in a limited way, (3) yes, but not fully, (4) fully compliant**
	Evaluation of One Health (OH) aspects **(collaboration across sectors/disciplines, knowledge integration, added value of OH approach, etc.)**	**(1) No OH aspects evaluated, (2) a few OH aspects evaluated, (3) many OH aspects evaluated, (4) consistens OH evaluation throughout**
**3—Pre-requisites before use**	**Required level of knowledge of users regarding surveillance, epidemiology and evaluation**	**(1) Specialist, (2) routine user, (3) basic, (4) no prior knowledge or experience required**
	**Training to get acquainted with the tool**	**(1) Impossible without, (2) highly recommended, (3) helpful but not required, (4) not necessary**
**4—Time and resources**	**Costs related to the access and use of the tool**	**(1) High recurring costs, (2) low recurring costs, (3) onetime costs, (4) no costs**
	Number of people in the evaluation team	**(1)** **>7 persons, (2) 5–7 persons, (3) 3–4 persons, (4) 1–2 person(s)**
	**Number of people to be interviewed**	**(1)** **>7 persons, (2) 5–7 persons, (3) 3–4 persons, (4) 1–2 person(s)**
	Duration of the evaluation process	(1) >2 months, (2) 1–2 months, (3) 1 week-−1 month, (4) <1 week
**5—Outputs**	Generation of actionable evaluation outputs	**(1) None; (2) outputs available, but not directly actionable, (3) available outputs partially actionable, (4) available outputs fully actionable**

^*^“Non-applicable” can also be used when scoring each aspect.

**Table 2:** Updated description of nine content themes^a^.

**Theme**	**Description (changes to the original are in bold)**
AMU and AMR	Questions that are specifically addressing the case of AMR (occurrence, prevention, or response) or AMU (recording, quantification and management)
Collaboration	Questions on the organization and functioning of the collaborative framework both for governance (including the inclusive participation of stakeholders and gender balance) and implementation of surveillance activities (including data and information exchanges, resources sharing, etc.)
Resources	Questions addressing human, material, and financial resources in terms of planning, allocation and availability. Questions on the training of human resources.
Output and use of information	Questions on integrated surveillance outputs that are provided to inform public and private stakeholders, their use to inform decision making, and the benefits from this use (expected, perceived, or measured)
Integration	Questions considering three levels of integration: 1. integration of knowledge (including that of information systems across organizations), 2. integration between sectors, professions and disciplines through a shared leadership, a shared decision making and planning process, the formulation of common goals, shared activities at the different stages of the surveillance process (data collection, communication, etc.) 3. integration at all the different decision-making levels (international, regional, national and local) and with the community
Adaptivity	Questions on any structural elements allowing the surveillance system to adapt and evolve **because of** internal and external changes. This may include governance mechanisms allowing the system to adapt (such as a steering committee with an effective feedback loop), as well as supporting tools (such as continuous learning programs, internal and external evaluation, monitoring of performance indicators)
Technical operations	Questions on technical features of the surveillance operations (surveillance design, data collection, laboratory capacities management of specimens, laboratory testing methods, data storage and management, data analysis and interpretation, communication, dissemination), their quality management (SOP, traceability), and the assessment of their performance (sensitivity and specificity)
**Impact**	**Questions related to all immediate, intermediate and ultimate changes (e.g., in knowledge, attitudes, practices, interventions, policies and health outcomes) that can be directly or indirectly attributed to the surveillance system. These changes can be positive and negative, intentional and unintentional**
**Governance** ^**b**^	**Questions related to accountability, coordination, participation, transparency and equity**.

^a^Scale to use: 1 = not covered, 2 = not well covered, 3 = more or less covered, 4 = well covered in line with Sandberg et al. (2).

^b^Governance was included as a separate theme in the case studies undertaken in Phase 1 of CoEvalAMR but is not appearing as a separate theme on the https://guidance.fp7-risksur.eu/welcome/decision-support/.

**Table 3:** Description of the four questions used for the SWOT-like approach, divided into the original and updated wording.

**Question**	**Topic**	**Original wording**	**Updated wording**
1	Strengths	Things that I liked, or that it covers well	The strengths of this tool are
2	Weaknesses	Things that I struggled with	The weaknesses of this tool are
3	Opportunities	Things people should be aware of when using this tool	The added value(s) of using this tool is (are)
4	Threats	Things that this tool is not covering or not good at covering	This tool might be criticized because of

During Phase 1, six tools were assessed using the described methodology, by applying them to eight national surveillance systems as country cases. The tools were: ATLASS (The Assessment Tool for Laboratories and AMR Surveillance Systems developed by the Food and Agricultural Organization (FAO) of the United Nations), ECoSur (Evaluation of Collaboration for Surveillance tool), ISSEP (Integrated Surveillance System Evaluation Project—now called ISSE), NEOH (Developed by the EU COST Action “Network for Evaluation of One Health”), PMP-AMR (The Progressive Management Pathway tool on AMR developed by the FAO), and SURVTOOLS (Developed in the EU FP7 project RISKSUR). An overall description of this work can be found in (2) whereas (3), described the Danish case study in detail. Moreover, a description of users' experience for each country case study can be found on the website of CoEvalAMR (https://guidance.fp7-risksur.eu/welcome/case-studies/). Some of these case studies consisted of full evaluations based on the tools used. In others, the focus was mostly on the tool, and therefore, the case study only included a superficial evaluation of the surveillance system.

We learned that some tools can be directly used to evaluate a given question, a surveillance component or a system. Such tools have a pre-defined set of steps that need to be conducted. Other tools are better described as frameworks, which provide a theoretical background and explanation as to how the evaluation should be designed. These frameworks guide users toward the most appropriate evaluation method based on the evaluation question and context. According to Calba et al. (4), a framework acts as a skeletal support for something being constructed. Hence, it is an organization of concepts that provides a focus for inquiry. In contrast, Calba et al. (4) define a tool as a process with a specific purpose. Therefore, a tool is used as a means of performing an operation or achieving an end. The ISSE is an example of a framework (5), whereas PMP-AMR is an example of a tool (6).

Among the tools and frameworks investigated, only the ISSE framework is dedicated specifically to the evaluation of integrated surveillance of AMU and AMR, outlining a logic model that can be used to conceptualize surveillance evaluations. Other tools, such as ATLASS and PMP-AMR, are designed specifically for AMU and AMR surveillance and management, NEOH for One Health initiatives in general, SURVTOOLS for surveillance in general, and ECoSur for integrated collaboration (2).

It was concluded that all tools investigated were suitable to evaluate relevant—but not necessarily all—aspects of integrated surveillance for AMU and AMR. Moreover, each tool has its specific purpose and consequently distinct advantages and drawbacks. This makes it important to define a clear evaluation question and objective to choose the right tool. We also learned that the complexity of the tool application appeared to be proportional to the comprehensiveness of the evaluation results. Moreover, governance and impacts of integrated surveillance for AMU and AMR were not fully covered by the assessment of the tools in Phase 1.

Hence, ample experience was collected regarding assessment of the tools and the developed methodology. It was concluded that the methodology worked, but the wording and definitions could be clearer, the evaluation coverage could be broadened, and the scoring system could be more standardized. It was also of interest to understand better the expectations of tool users. Moreover, we wanted to compare the CoEvalAMR methodology with the assessment process used in the newly published Surveillance and Information Sharing Operational Tool (SISOT) (7), developed by the Tripartite (FAO/WHO/WOAH) of the United Nations (UN). These aspects have been dealt with in Phase 2 of the CoEvalAMR project, which runs from 2021 to 2023. The objective of this paper is to present the updated methodology, including an example showing the changes, as well as the considerations behind the update.

## Materials and methods

In spring 2021, monthly virtual meetings began in the network, allowing members to convene and discuss how to update the methodology. A common document was set up enabling all members to provide comments and suggestions, which were subsequently discussed with the aim of obtaining consensus. This process continued until autumn 2022. Three elements were discussed: (1) lessons learned from using the initially developed methodology, (2) an analysis of expectations of tool users, and (3) the assessment process used in SISOT. Regarding lessons learned, the approach was a brainstorm in the groups' monthly meetings.

Regarding expectations of the tool users, we considered the results of a survey by Rüegg et al. (8). The survey was conducted in Phase 1 of CoEvalAMR to gather information on evaluation of existing or planned AMU and AMR surveillance systems and people's use of available evaluation tools, as well as their expectations on tools. An analysis of the 23 answers received was undertaken. We studied and discussed how we could best make use of these results to update the CoEvalAMR methodology.

Further, we looked at the list of functional aspects and content themes in SISOT and assessed if any of these would be of value for the update of the methodology. We also studied the definitions, use of scales, and visual appearance. Based on discussions in the CoEvalAMR network group, we aimed at identifying additional functional aspects, which would make the description of the individual tools more complete.

Finally, the updated methodology was tested using a case study undertaken as part of our network. Here, ECoSur was applied to the French surveillance system for AMU, AMR and antimicrobial residues in humans, animals and the environment (9). The overall objective was to evaluate the degree and quality of multisectoral collaboration within the surveillance system. In accordance with the aim of ECoSur, the focus was on evaluating the organization, functioning and functionalities of collaboration taking place in the French multi-sectoral surveillance system. The tool is available online (https://survtools.org/wiki/surveillance-evaluation/doku.php?id=quality_of_the_collaboration), for more information about ECoSur, please see Bordier et al. (10).

## Results

### Lessons learned from use of the initially developed evaluation methodology

The lessons learned on the methodology in Phase 1 of the network were the following:

- It takes time to make an assessment, as this requires first to get acquainted with the tool, and next to collect the necessary information and thereafter apply the tool.
- Inevitably, there is a high level of subjectivity in the assessment process, especially when it comes to developers assessing their own tools, but also to users, who are not acquainted with the tool.
- Clear definitions for all functional aspects and content themes—including the individual scores—are needed to ensure common understanding and harmonized scoring across future assessors.
- A justification is required along with the semi-quantitative scores to ensure meaningful interpretation because a specific score can be given for different reasons.
- To illustrate variation between assessors, an approach should be developed to combine the scores from different assessors/different case-studies.
- Regarding the SWOT-analysis (Table 3), the question related to opportunities was misinterpreted by some of the tool evaluators, who referred to negative aspects of the tool instead of positive aspects.

### Analysis of expectations of tool users

The analysis of the 23 answers to the questionnaire undertaken by Rüegg et al. (8) showed that the respondents emphasized the following:

- The tools should provide clear results and evidence of data integration quality that can be used with confidence in research or to inform decision making.
- Standardized guidance should be available regarding which tool to use, depending on the evaluation needs.
- There should be an increased awareness of the different integrated evaluation tools available to stakeholders and in which contexts each tool could be used.
- It should be possible to undertake different levels of evaluation from superficial to deep, to enable, e.g., a rapid “general overview” evaluation with a more detailed evaluation of selected components.
- Standardized evaluation attributes and measurements across all evaluation tools would enable comparisons to be made between evaluations that use different tools.
- Standardized evaluation methods should enable evaluations that are comparable between different components.
- All tools should be free and easy to use with services available to guide users.
- Clear and easy to use tools would help to minimize bias and subjectivity of the person evaluating the system.
- There should be an opportunity to get assistance from an expert to discuss the different tools available and how and when to use them.

Essentially, people would like to see a one-stop shop with standardized tools that are flexible and easy to use. This does not sound realistic, but it puts attention to the requirement for an approach which is simple, transparent, and with clear definitions. It also means that there should be a balance between the more detailed parts of the evaluation and the general overview.

### Comparison between the CoEvalAMR methodology and SISOT

The SISOT has recently been developed by the Tripartite of the UN to support national authorities in establishing or strengthening their coordinated multisectoral surveillance and information sharing for zoonotic diseases (7). SISOT can be used for identifying useful tools and resources for creating, implementing, and/or maintaining coordinated surveillance capacity, and information sharing platforms. The intention is to collect a repository of tools and resources to help users in identifying the most suitable tools and resources. Hence, the objective is like the work undertaken in CoEvalAMR which is focusing on AMU and AMR surveillance, but for a wider context as SISOT is targeting all zoonotic diseases and health threats shared between different domains.

The SISOT Evaluation Matrix describes a tool or resource using a standardized set of criteria that can be used to evaluate whether it is fit for a given purpose. The matrix can be applied to all tools and resources, which can assist in completing any step toward creation of a coordinated zoonotic surveillance system. The criteria are used to identify the strengths and weaknesses in an objective and unbiased way. There are nine categories of criteria: (1) accessibility, (2) language, (3) data needs and management, (4) data analysis and interpretation, (5) ease of use, (6) flexibility, (7) acceptability, (8) One Health, and (9) tool impact. For each category, the evaluation must address a series of pre-defined questions. There are between 3 and 10 questions per category, and for each question a scale of 1–5 is used depending on the situation observed. Radar diagrams are used to provide a graphical presentation of the results of scoring, illustrating the scores on nine different axes corresponding to each category. An evaluation criteria score is given up to a maximum of 100%. FAO has been undertaking country pilots using the SISOT Evaluation Matrix (7).

Based on the investigation of the SISOT Evaluation Matrix and discussions in the CoEvalAMR network, we identified that the addition of the following functional aspects would make the CoEvalAMR methodology more complete:

- Type of approach: framework or tool,
- Scoring-system method (quantitative, semi-quantitative or qualitative),
- Required level of knowledge of users regarding surveillance, epidemiology, etc.
- Required training to be acquainted with the tool,
- Coverage of the tools: human domain, animal domain, environmental, and food domain and combinations thereof,
- Coverage of gender aspects,
- Accessibility, and
- Languages in which the tool is available.

### The updated CoEvalAMR methodology

The following updates were made on the existing CoEvalAMR methodology: First, the description of the case study was updated (Supplementary Table S1). Then, a general description of the tool, based on 10 functional aspects, was added (Supplementary Table S2). One of these aspects was gender equity. The list of functional aspects to be scored is presented in Table 1, along with the scoring system, defined in more detail than before. The functional aspects are now classified into five groups. Similarly, the updated content themes used to describe the scope of the tool are presented in Table 2, along with the original definition and the updated definition applied in Phase 2. Two new themes were included: governance and impact. The scoring system for the content themes was maintained, implying a four-tiered scale, where 1 = not covered, 2 = not well covered, 3 = more or less covered, 4 = well covered, in line with Sandberg et al. (2). The challenges related to the four SWOT questions was solved by using the words “strengths,” “weaknesses,” “opportunities,” and “threats” (Table 3).

**Figure 1:**
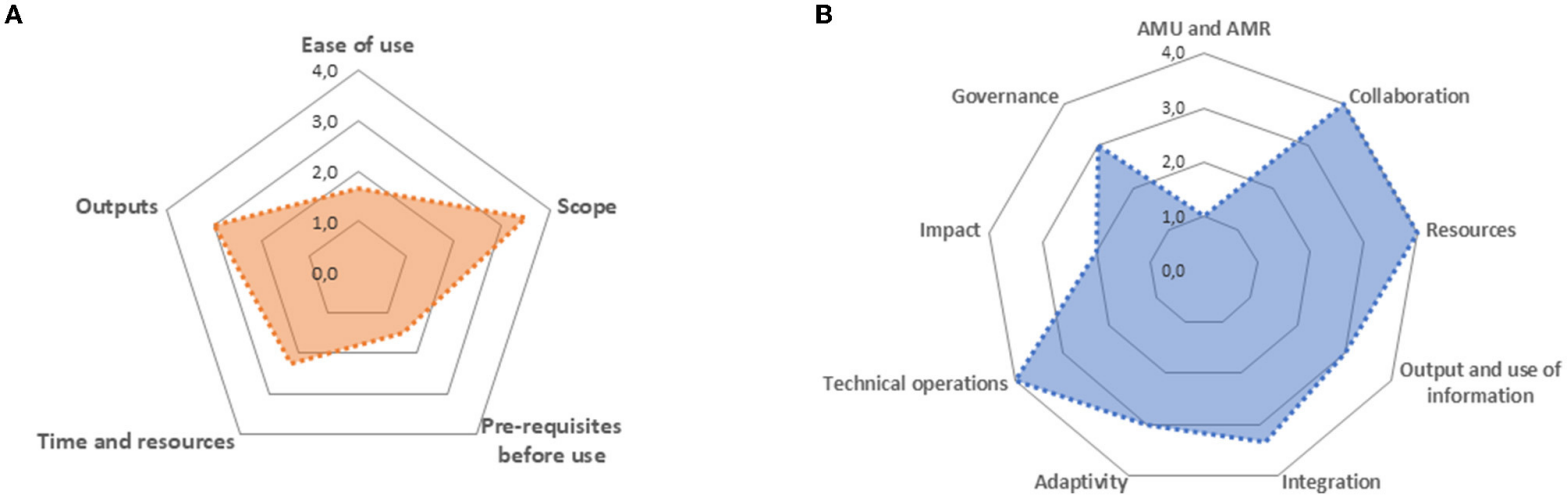
Radar diagrams depicting graphically the scoring of the functional aspects **(A)** and the content themes **(B)**, based upon a French case study using ECoSur. Source: (https://guidance.fp7-risksur.eu/case-studies/).

Visualization of the results was improved by developing radar diagrams as a way of presenting the scoring of functional aspects and content themes. An example is given in Figure 1A for the functional aspects and in Figure 1B for the content themes. Nine axes were judged as the maximum number of axes, which could be used while having a readable graphical output. Therefore, some of the functional aspects were combined. Table 3 contains the original four questions used for the SWOT-like analysis along with the revised questions. The templates are now combined in an Excel matrix, which can be found on the website of CoEvalAMR (https://guidance.fp7-risksur.eu/case-studies/).

The Excel matrix using the revised methodology was pilot tested as part of the French case study on the evaluation of collaboration within the French surveillance system for AMR, AMU and antimicrobial residues using ECoSur (11). The completed matrix can be consulted on the CoEvalAMR case studies repository (Please see Case 9 on https://guidance.fp7-risksur.eu/case-studies/). Briefly, the assessment demonstrated that despite ECoSur being somewhat difficult to use (collection of complex data and need for prior knowledge/training before use), it covered a large part of One Health aspects and generated actionable outputs (Figure 1A). In addition, most content themes identified by the CoEvalAMR consortium as relevant to the evaluation of integrated surveillance of AMU and AMR were covered by ECoSur, with the exception of AMU/AMR specific aspects (ECoSur being a generic tool) and impacts (Figure 1B).

## Discussion

In Phase 1 of the CoEvalAMR network project, it was found that the users scored the individual functional aspects and content themes in a slightly subjective way. As the project progressed, a higher degree of consensus arose regarding interpretation of the methodology, including the way of scoring (2). Moreover, we discovered that the third question in the SWOT analysis was misunderstood by some of the users. We expect that with the update of the methodology, subjectivity will be reduced. Similarly, the likelihood of misunderstanding the questions will be lower.

The importance of considering gender and equity to tackle AMR has been underlined by the WHO (12, 13) but is currently rarely integrated into surveillance system evaluation. As explained by WHO, unless we think about how AMU and AMR affect men and women and different groups in society in their day-to-day lives at home, work and in their communities, we may inadvertently design programs that fail to address what matters. Hereby, effectiveness may be reduced, and impacts lost, and we may even contribute to gaps and inequities (12). As a first step toward enhancing the inclusion of this aspect, we have added consideration of gender to the list providing a general description of the tools (Supplementary Table S2). Still, we foresee a discussion on how to assess and evaluate gender aspects and other equity issues of importance for AMU and AMR. These issues may become part of a future Phase 3 of our network. Here, chapter 4 in the Handbook for Evaluation of One Health may provide inspiration for the next steps to take (14).

The respondents of the questionnaire survey undertaken as part of Phase 1 of CoEvalAMR pointed to the need for standardization of tools (8). In response to that, we have focused on standardizing our methodology by introducing clearer definitions and scales. The question arises as to which extent further standardization of our methodology is needed. It may be argued that standardization is an essential requirement in academia, but a less important issue for persons involved with the human health and veterinary authorities, where the process initiated by the tool would be more important than the tool itself. Moreover, the intention is not to compare tools, but to describe the tools to such an extent that the future users will be guided in choosing the right tool for their purpose.

According to the survey, the users prefer tools that are easy to use, without much need for preparation or training (8). The question is how this can be operationalized. Grants are usually targeting the development of tools, whereas limited resources are available for supporting their uptake and long-term maintenance. Moreover, the results of simple evaluations may not be sufficiently valuable. Still, it is relevant to discuss the balance between required training, allocated resources, details and overview. To address this, the intended outcome of the evaluation becomes crucial. This reiterates the need for careful description of the evaluation purpose before choosing the evaluation tool.

In our updating of the methodology, we have been inspired by the SISOT matrix developed by the Tripartite. The SISOT matrix is very detailed and can be used for evaluating different kinds of tools and resources for any zoonotic risk-reducing activities. The questions and possible ways of answering show how well-developed the SISOT matrix is. Our revised CoEvalAMR tool is targeting integrated surveillance for AMU and AMR. Based upon our own experience as well as the French case study (11), the CoEvalAMR methodology appears simpler and quicker to use than the SISOT matrix, while it still contains most of the elements that form part of the SISOT matrix. In conclusion, each approach was developed for its own objectives and has its value.

The case studies reported by Sandberg et al. (2) and Nielsen et al. (3) and the French case study (9, 11) covered both multi-component and single component surveillance systems. Multisectoral means that more than one sector is working together in a joint program or response to an event. Similarly, multidisciplinary means collaboration across several disciplines. Taking a One Health approach means that all relevant sectors and disciplines are involved (15). However, it does not imply that all sectors must work together and at all stages of surveillance. The key regarding the degree of integration is relevance. For example, the Competent Authority may need AMU and AMR data in animals and humans to evaluate the effect of a ban on use of a specific kind of antimicrobial in agriculture. However, data on AMR from the environment may not be needed. In contrast, if we are trying to understand the spread of AMR in the environment, data about AMU and AMR are needed from all three sectors. The methodology we have developed is useful to provide an overview of the advantages and disadvantages of the tool investigated, irrespective of whether the tool was used for evaluation of an integrated or non-integrated surveillance system.

Evaluation of One Health surveillance is an active field, and there is a growing number of evaluation tools becoming available. The Canadian One Health Evaluation of Antimicrobial Use and Resistance Surveillance (OHE-AMURS) tool is an example of such a new tool. It has been created to evaluate progress toward integrated, One Health surveillance of AMU and AMR while focusing among others on policy and programme sustainment (16). In Sandberg et al. (2), six tools were retained for evaluation. The ambition in Phase 2 of CoEvalAMR is to apply the updated evaluation methodology to other tools, in accordance with the needs or interests of the network members. The French case study is an example of this. It showed that there is a diversity of individual surveillance programs in France (9). This makes it difficult to get an overview of the surveillance system and its level of integration (11). The ECoSur evaluation provided this overview and helped to identify recommendations, which were shared with policy makers to improve One Health collaborations within the French system for surveillance of AMR, AMU, and AM residues (11).

An ongoing common activity in WG4 of CoEvalAMR is an evaluation of the OH-EpiCap tool, which is under development by the MATRIX consortium, funded by the One Health European Joint Program (17). In a common paper about OH-EpiCap, it will be investigated how we can combine the scores of the different assessors and case studies in a way which ensures that the variation is reported.

Other persons involved in surveillance evaluation are welcome to make use of our methodology. Moreover, the tool developers can get valuable information from our case studies for further improvements of their tools.

## Conclusion

The CoEvalAMR evaluation methodology is developed with a focus on the users’ experience. It is free to use, simple and easy to work with. It has been updated to improve clarity, broaden the evaluation coverage, increase the standardization, and improve the visual appearance. The update was based upon experience from the CoEvalAMR network group from applying the methodology using country case studies, a questionnaire focused on the users' needs as well as a comparison with SISOT Evaluation Matrix developed by the Tripartite.

## Data availability statement

The raw data supporting the conclusions of this article will be made available by the authors, without undue reservation.

## Author contributions

LA took the initiative to update the methodology and headed the revision process through a series of meetings in WG4 of the CoEvalAMR network. Based upon the inputs from the group, LA drafted the first version of the paper, which was commented by all authors. The excel version of the CoEvalAMR tool was developed by PM. LC is responsible for the French case study. All authors read and approved the final version of the manuscript.
