# Supplementary material for: Capturing systematically users' experience of evaluation tools for integrated AMU and AMR surveillance
Source: Front Vet Sci. 2023 Mar 24;10:1107122. doi: 10.3389/fvets.2023.1107122 (PMC10081675; doi:10.3389/fvets.2023.1107122)
Supplement: Supplementary file 1 [file Data_Sheet_1.docx]

**Supplementary Material**

**Table S1 - General information about the case study**

Name of evaluation tool: ___________________________________________________________

Name of surveillance system used in case study: _________________________________________

Country of implementation: _______________________________________________________

Surveillance component or system covers (tick at least one):

AMU   AMR   Both   Other please describe: ____________________________

Please describe: What is covered by (part of) the component or system evaluated (tick at least one):

Hospitals Human primary care Human long-term care

Livestock   Aquaculture   Bees   Green environment   Aquatic environment

Food chain   Companion animals   Equidae   Camelids and Deer   Wildlife

Other please describe: _____________________________________________________

Objective(s) of evaluation (tick at least one):

Performance   Infrastructure   Functionality   Operations   Collaboration

One Health-ness / the strength of One Health   Impact

Other please describe: _____________________________________________________

Main results of evaluation: __________________________________________________________

Time period for evaluation: _________________________________________________________

Name(s) of evaluator(s): ________________________________________________________

Affiliation of evaluator(s): _______________________________________________________

Acquaintance of the evaluator(s) with the tool (tick at least one):

Owner   Developer   User without involvement in development or ownership of tool, but developer did facilitate the evaluation process   Other please describe:______________

Citation of work, if published:________________________________________________________

Contact (email address):_______________________________________________________

**Table S2. Aspects to fill in as a brief description of the tool**

| **Additional aspects to describe the tool** | **Possible outcomes** |
| --- | --- |
| Sector covered | Human, animal, environmental, and food domain and combinations thereof |
| Gender aspects covered  Languages | Yes/no  Number of languages for which the tool is developed |
| Type of approach | Tool or framework |
| Type of scoring system | Quantitative, semi-quantitative or qualitative |
| Use of a stepwise approach to scoring the surveillance system  Purpose of the tool | Yes/no  Performance, collaboration, OH-ness, planning, etc. |
| Target users | Scientists, donors, surveillance system coordinators, etc. |
| Resources available about the tool | Scientific articles, reports, tool itself, repository of case studies |
| Accessibility | Is the tool freely available online or not |
